# Human fascioliasis endemic areas in Argentina: multigene characterisation of the lymnaeid vectors and climatic-environmental assessment of the transmission pattern

**DOI:** 10.1186/s13071-016-1589-z

**Published:** 2016-05-27

**Authors:** María Dolores Bargues, Jorge Bruno Malandrini, Patricio Artigas, Claudia Cecilia Soria, Jorge Néstor Velásquez, Silvana Carnevale, Lucía Mateo, Messaoud Khoubbane, Santiago Mas-Coma

**Affiliations:** Departamento de Parasitología, Facultad de Farmacia, Universidad de Valencia, Av. Vicente Andrés Estellés s/n, 46100 Burjassot, Valencia Spain; Facultad de Ciencias de la Salud, Universidad Nacional de Catamarca, Maestro Quiroga 1ra. Cuadra, CP 4700 San Fernando del Valle de Catamarca, Argentina; Hospital Municipal de Infecciosas “Dr. Francisco Javier Muñiz”, Uspallata 2272, CP 1282 Ciudad de Buenos Aires, Argentina; Instituto Nacional de Enfermedades Infecciosas - ANLIS “Dr. Carlos G. Malbrán”, Av. Vélez Sársfield 563, CP 1281 Ciudad de Buenos Aires, Argentina; Consejo Nacional de Investigaciones Científicas y Técnicas (CONICET), Av. Rivadavia 1917, CP 1033 Ciudad de Buenos Aires, Argentina

**Keywords:** Human fascioliasis, *Lymnaea neotropica*, *Lymnaea viator*, Vectors, rDNA, mtDNA, Morphometry, Climate, Environment, Argentina

## Abstract

**Background:**

In South America, fascioliasis stands out due to the human endemic areas in many countries. In Argentina, human endemic areas have recently been detected. Lymnaeid vectors were studied in two human endemic localities of Catamarca province: Locality A beside Taton and Rio Grande villages; Locality B close to Recreo town.

**Methods:**

Lymnaeids were characterised by the complete sequences of rDNA ITS-2 and ITS-1 and fragments of the mtDNA 16S and *cox*1. Shell morphometry was studied with the aid of a computer image analysis system. Climate analyses were made by nearest neighbour interpolation from FAO data. Koeppen & Budyko climate classifications were used. De Martonne aridity index and Gorczynski continentality index were obtained. Lymnaeid distribution was assessed in environmental studies.

**Results:**

DNA sequences demonstrated the presence of *Lymnaea neotropica* and *L. viator* in Locality A and of *L. neotropica* in Locality B. Two and four new haplotypes were found in *L. neotropica* and *L. viator*, respectively. For interspecific differentiation, ITS-1 and 16S showed the highest and lowest resolution, respectively. For intraspecific analyses, *cox*1 was the best marker and ITS-1 the worst. Shell intraspecific variability overlapped in both species, except maximum length which was greater in *L. viator*. The desertic-arid conditions surrounding Locality A, the semiaridity-aridity surrounding Locality B, and the very low yearly precipitation in both localities, are very different from the typical fascioliasis transmission foci. Lymnaeids are confined to lateral river side floodings and small man-made irrigation systems. Water availability only depends on the rivers flowing from neighbouring mountains. All disease transmission factors are concentrated in small areas where humans and animals go for water supply, vegetable cultures and livestock farming.

**Conclusions:**

The unusually high number of DNA haplotypes and the extreme climate unsuitable for *F. hepatica* and lymnaeid development, demonstrate that the transmission foci are isolated. Seasonal transmission may depend on the timely overlap of appropriate temperature and river water availability. Lymnaeids and *F. hepatica* have probably reached these localities by livestock introduction. DNA differences regarding other populations of *L. neotropica* and *L. viator* in Argentina suggest an introduction independent from the spreading movements which allowed these two lymnaeids to expand throughout the country.

**Electronic supplementary material:**

The online version of this article (doi:10.1186/s13071-016-1589-z) contains supplementary material, which is available to authorized users.

## Background

Fascioliasis is a foodborne trematodiasis caused by species of the genus *Fasciola*. This disease affects livestock almost everywhere and humans in many countries of Europe, Africa, Asia, the Americas and Oceania [1, 2], where there is tradition of eating uncooked vegetables carrying infective metacercariae [3]. Fascioliasis is emerging in many countries, with progressive detection of new human fascioliasis endemic areas and an increasing number of human case reports. This emergence phenomenon has partly been related to climate change [4] and also global change aspects [5] such as anthropogenic modifications of the environment [6], travelling [7] and import/export of livestock [2]. These changes appear to be related to the high dependence of both fasciolid larval stages and their freshwater lymnaeid snail vectors on climatic and environmental characteristics [8–10]. The increasing importance of human fascioliasis also relies on pathogenicity and immunity. Thus, this disease appears to be pronouncedly complicated including diagnosis difficulties [11] and a great morbidity impact on children in long-term infections, such as in human fascioliasis endemic areas [12–15]. The clinical complexity of the symptoms and syndromes due to the capacity of the flukes to affect vital organs other than the liver, giving rise to important sequelae and even death, add concern about human fascioliasis [16].

Fascioliasis is transmitted by freshwater lymnaeid snails which show marked specificity according to *Fasciola* spp. [17]. *Fasciola hepatica*, distributed throughout all continents, is mainly transmitted by species of the “fossarine” or *Galba*/*Fossaria* group [18, 19]. *Fasciola gigantica*, present in large regions of Africa and Asia, is mainly transmitted by species of the *Radix* group [20]. Another vector species is *Pseudosuccinea columella*, a species with global distribution, able to transmit both *Fasciola* spp. and above all related to animal infection [21]. Other lymnaeid groups include species which may only act as secondary or sporadic vectors and without epidemiological importance except under special circumstances [22, 23].

Although livestock species play an important reservoir role, transmission studies have shown that the metacercarial infective stage from different origins, such as sheep, cattle, pig and donkey, represent similar infectivity sources [24, 25]. On the contrary, the susceptibility of a given lymnaeid species to fasciolid infection represents a crucial factor in establishing not only the geographical distribution of the disease in both animals and humans, but also prevalences and intensities due to more or less appropriate ecological characteristics (population dynamics, anthropophylic characteristics, type of water bodies, etc.) of the different lymnaeid vector species. This is why different lymnaeid species appear linked to the different transmission patterns [26] and epidemiological scenarios [27] of this very heterogeneous disease in humans [2]. The continental differences in lymnaeid faunas also explain that in the Americas fascioliasis is only caused by *F. hepatica*, due to the absence of lymnaeids of the genus *Radix* which act as vectors of *F. gigantica* [20]. Similarly as in other vector-borne diseases, this relationship supports the use of lymnaeids as biomarkers of the disease at both local and large scales [28]. Distribution, both in space (latitudinal, longitudinal and altitudinal) and time (seasonal, yearly), of fascioliasis depends on the presence and population dynamics of the specific mollusc species, which in turn is linked to the presence of the appropriate water bodies and on adequate climate characteristics enabling fluke development.

Within the several human fascioliasis regions known [2], the Americas stand out due to the human endemic areas described in many countries. High prevalences and intensities of *F. hepatica* in humans have been reported in Andean countries such as Bolivia [29–33], Peru [34–36] and Chile [37, 38]. Human endemic areas have also been described, although with lower prevalences and intensities, in Central America [39] and recently also in North America [40]. Several human cases have also been reported from altitude areas of Ecuador [41], Colombia [21] and Venezuela [28]. In Uruguay and Brazil, human reports only concern sporadic and isolated cases. In the Caribbean region, human fascioliasis mainly poses problems in Cuba, where patients are continuously diagnosed [42, 43] even in high numbers [44], and Haiti [45]. Puerto Rico may still be considered an area of risk for human infections considering the epidemiological situation in the past [46].

In Argentina, physiography, climate, animal prevalence of *F. hepatica* and lymnaeid species composition are similar to those of countries where human fascioliasis endemic areas are present, such as Bolivia, Peru and Chile. A retrospective analysis highlights that human fascioliasis may have been overlooked in the past and its real epidemiological situation in high risk rural, mainly altitudinal areas, may currently be underestimated. Moreover, a long delay in diagnosis (almost 3.5 years on average) and high lithiasis proportion suggest that many patients are frequently overlooked and pose a question mark about fascioliasis detection in the country [47]. The recent detection of lymnaeid vector species such as *G. truncatula* [48, 49] and *L. neotropica* [19, 50], well-known to be responsible for high prevalences and intensities of human fascioliasis in neighbouring and closely located countries such as Bolivia and Peru [51], adds concern to this question.

In Argentina, several outbreaks presenting typical food-borne characteristics appear related to the most common risk factor: ingestion of watercress naturally growing along the river- and stream-beds picked during recreational, weekend or vacation activities. Many of these field excursions are undertaken by a family or as a group activity. This explains why family outbreaks have been noted to be common, whereas isolated cases seem to be rare in the country [52]. Outbreaks described in eleven families involving a total of 63 people [52–58] including a maximum of up to 15 family members affected at once [52], are good examples.

However, different epidemiological situations were recently detected in two areas of this country as a result of two serological surveys performed with an ELISA test for the detection of anti-recombinant procathepsine L1 of *F. hepatica* (Fh-rproCL1) antibodies [59, 60]. In the area of Arroyo El Juncal, La Toma, province of San Luis, between 500 and 600 m altitude, the prevalence was 11.90 % in a total of 42 human subjects (2 infected out of 23 males and 3 out of 19 females) serologically analysed [61]. This corresponds to a human hyperendemic situation according to the WHO classification [27]. In the same El Juncal area, 5.26 % out of 19 livestock species proved to be infected after coprological analysis (only in cattle; sheep and horses were not infected) and a surprisingly high prevalence (61.76 % out of 34 snails) was detected by sequencing a 447 bp long fragment of the mtDNA *cox*1 gene of *F. hepatica* [61] in snails identified as *Lymnaea viator* (= *L. viatrix*; for nomenclature see [38]).

In the area of the villages of Taton and Rio Grande, Department of Tinogasta, province of Catamarca, at an altitude of 1,630 m, the situation appears to be worse. A total of 54 subjects proved to be positive out of 148 serum samples analysed (17 positive out of 61 males and 37 out of 87 females) [62]. This means a very high prevalence of 36.5 %, at the level of the highest hyperendemic situations known in the Northern Altiplano of Bolivia [30–33] and Peru [34], as well as the valley of Cajamarca also in Peru [36]. Indeed, a public health problem posed by fascioliasis throughout the Argentinian province of Catamarca was already suspected in preliminary studies which allowed for the detection of ten serologically positive patients (without eggs in stools) in the locality of Recreo in 2002 [63].

The present study aims to (i) identify the lymnaeid vector species involved in both endemic areas of Taton-Rio Grande and Recreo by means of molecular tools; (ii) characterise these lymnaeids by their shell morphometry and genital anatomy; and (iii) analyse their habitats and respective relationships with climatic factors to assess the transmission pattern of fascioliasis in the endemic areas. For the molecular characterisation, both nuclear ribosomal DNA and mitochondrial DNA markers are sequenced and subjected to phylogenetic analyses. Snails are studied by traditional malacological methods to ascertain whether morphology may allow species differentiation. In the Americas, except *P. columella*, all lymnaeid species involved in the transmission belong to the *Galba*/*Fossaria* group. Species differentiation within *Galba*/*Fossaria* is crucial given (i) their different disease transmission capacities to humans; (ii) the difficulties in species differentiation due to their pronounced morphological similarities; and (iii) the fact that there is a snail species of this group which, although usually present in high transmission areas, does not transmit [18]. For the climatic assessment, a deep analysis by different climate classifications and indices is made additionally for the study of the climatic factors related to fascioliasis transmission, given the very peculiar, isolation characteristics of the transmission foci [64, 65].

## Methods

### Lymnaeid snails

**Fig. 1:**
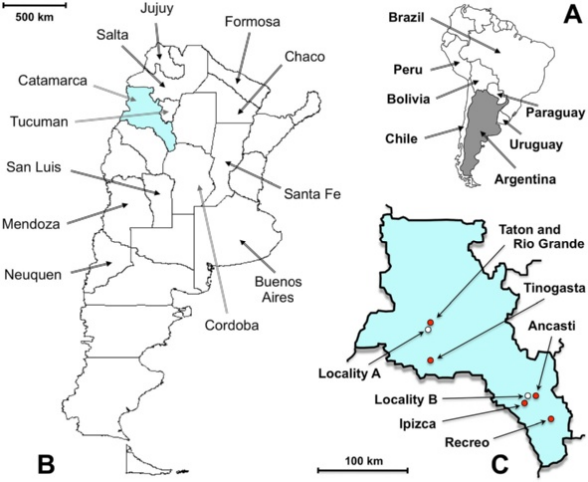
Maps showing the transmission foci studied where the lymnaeid snails were collected. **a** Location of Argentina in South America. **b** Location of the province of Catamarca inside the country (names of provinces only in those where human infection has been diagnosed). **c** Location of the sampling Locality A (*white circle*) beside the human fascioliasis endemic villages of Taton-Rio Grande and Tinogasta (*red circles*), in the Department of Tinogasta, and sampling Locality B (*white circle*) beside the villages of Ipizca and Ancasti and close to the human fascioliasis endemic town of Recreo (*red circles*), in the Department of La Paz

The snail specimens studied were collected in the field, from lymnaeid populations present in two geographical areas of the Province of Catamarca (Fig. 1):

- Locality A: two neighbouring fertile plains where livestock go for drinking (27°29′52.62″S, 67°35′53.51″W; altitude 1,630 m), only 300 m far from the river, in the way to the small villages of Taton (1860 m altitude) and Rio Grande (2825 m) (Departament of Tinogasta). The zone comprises two villages, Taton of about 150 inhabitants living in dwellings spread throughout 2 km at the two banks of the river coming down from the smaller Rio Grande of 40 inhabitants and several more or less scattered dwellings distributed on the hills (called “Puestos”); human infection by *F. hepatica* has been reported from inhabitants of both villages [62];
- Locality B: an artificial dyke or dam (28°49′08.83″S, 65°32′20.00″W; altitude 944 m) beside the village of Ipizca (Department of Ancasti) and close to the town of Recreo (Department of La Paz) where human infection has also been reported [63].

For molecular analyses, live snail specimens were fixed in 70 % ethanol for DNA extraction procedures. A total of 50 lymnaeid specimens from Locality A and six specimens from Locality B were collected and used for molecular and morphometric studies.

### Molecular techniques

#### DNA markers

The molecular characterisation of the snails has been made by DNA sequencing of the complete nuclear ribosomal DNA (rDNA) spacer markers ITS-2 and ITS-1 and fragments of the mitochondrial DNA (mtDNA) gene markers 16S rDNA and *cox*1. The usefulness of these markers has been already proven for invertebrates in general [66] and for the classification of the lymnaeid species and the comparative assessment of the intraspecific variability of their populations in many countries of Latin America [17–21, 28], also including Argentina [48–50].

#### DNA extraction

DNA extraction was performed individually from the head-foot tissue of each snail with phenol-chloroform and DNA was precipitated with ethanol. The snail specimens were suspended in 400 μl of lysis buffer (10 mM Tris–HCl, pH 8.0, 100 mM EDTA, 100 mM NaCl, 1 % sodium dodecyl sulfate SDS) containing 500 μg/ml Proteinase K (Promega, Madison, WI, USA) and digested for 2 h at 55 °C with alternate shaking each 15 min. The procedure steps were performed according to methods outlined previously [19, 20]. The pellet was dried and resuspended in 30 μl sterile TE buffer (pH 8.0); this suspension was stored at −20 °C until use.

#### Sequencing of rDNA and mtDNA markers

The four DNA markers were PCR amplified independently for each specimen and each PCR product was sequenced for a bona-fide haplotype characterisation. The complete rDNA spacers ITS-2 and ITS-1 were amplified using previously described primers [2, 19, 20, 48, 49]. The target fragments of the 16S and *cox*1 genes were amplified by PCR using a set of universal primers [67, 68]. Amplification procedures and thermal cycler conditions for each one of the DNA markers were carried out in a Mastercycle ep*gradient* (Eppendorf, Hamburg, Germany), as previously described [2, 18].

PCR products were purified using the Ultra Clean™ PCR Clean-up DNA Purification System (MoBio, Solana Beach, CA, USA) according to the manufacturer’s protocol and resuspended in 50 μl of 10 mM TE buffer (pH 7.6). The final DNA concentration was determined by measuring the absorbance at 260 and 280 nm on an Eppendorf BioPhotometer (Hamburg, Germany).

Sequencing was performed on both strands by the dideoxy chain-termination method and carried out with the Taq dye-terminator chemistry kit on an Applied Biosystems 3730 DNA Analyzer (Applied Biosystems, Foster City, CA, USA) using the PCR primers*.*

#### Sequence analyses

Sequences were aligned using CLUSTALW2 [69] in MEGA 6.0.6 [70] using default settings. Minor corrections for a better fit of nucleotide or indel correspondences were made in the cases of the ITS spacers. Homologies were performed using the BLASTN programme from the National Center for Biotechnology information website (http://www.ncbi.nlm.nih.gov/BLAST). As the systematics of the South American lymnaeids belonging to the *Galba*/*Fossaria* group is controversial and specimen identification cannot be made with the use of morphological criteria alone [19, 50], sequence comparisons were made using all ribosomal and mitochondrial sequence data for molluscs available in the GenBank database.

#### Phylogenetic inference

For phylogenetic analyses, ITS-1 and ITS-2 combined haplotypes were used, given that the *Galba*/*Fossaria* group includes species originating in both the Old and New Worlds. The division of the Gondwana is estimated back to around 100 million years [71]. This long evolutionary period indicates the inappropriateness of using mtDNA markers for the phylogenetic assessment, due to the well-known problem of mtDNA nucleotide saturation distorting the phylogenetic information [66]. Details of combined haplotypes and GenBank codes of the sequences of lymnaeid species used in the phylogenetic analyses, others than the new ones obtained for *L. neotropica* and *L. viator* in the present study, may be found in a previous analysis [18].

The phylogenetic analyses were performed with maximum likelihood (ML) and distance-based (Neighbour Joining) (NJ) methods implemented in MEGA 6.0 [70] and PAUP 4.0 b10 [72], respectively. The best-fitting substitution model was determined with jModeltest version 0.1.1 [73] based on the corrected Akaike’s information criterion (AICc) [74], which led to the selection of the Kimura 2-parameter model with a gamma-distributed rate heterogeneity among sites, with five rate categories (K2 + G). The initial tree for the heuristic search was automatically obtained by applying NJ and BioNJ algorithms to a matrix of pairwise distances estimated using the Maximum Composite Likelihood (MCL) approach.

To provide an assessment of the reliability of the nodes in the tree, a bootstrap analysis using 1,000 replicates was performed with two types of search, Neighbour-Joining and “Fast” stepwise-addition in PAUP. Moreover, a Bayesian approach was applied to reconstruct the phylogeny of the concatenated dataset in MrBayes 3.1.2 [75], using the same evolutionary model as above. Posterior probabilities were estimated by a 1,000,000 generations (four chains) and trees were sampled every 100 generations. The first 1,000 trees sampled were ruled out (“burn-in”), and clade posterior probabilities (PP) were computed from the remaining trees. The intergenic region sequence (GenBank AY030361) [76] including both ITS spacers of the planorbid *Biomphalaria pfeifferi*, was used as the outgroup.

#### DNA haplotype nomenclature

The haplotype (H) terminology used for the sequences obtained follows the previously described standard nomenclature proposed for lymnaeid snails [2, 17, 48]. It should be noted that haplotype codes are only definitive in the case of complete sequences; when dealing with fragments or incomplete sequences, haplotype codes are provisional.

### Morphometric study

Only shells from simultaneously molecularly assessed lymnaeid specimens were used (*L. neotropica*: *n* = 34; *L. viator*: *n* = 16) for the morphometric study and comparative analyses. The comparative morphometric characterisation of the lymnaeid shell may be a feature of interest [18, 19, 77], as it may help health officers in their field work in the case that different lymnaeid species coexisting in the same endemic area can be distinguished morphologically.

Snail shells were measured according to traditional malacological methods [77, 78], using a computerised image-analysis system (CIAS) [79, 80]. This system is based on a DXC-930P colour video camera (Sony DXC-930P, Tokyo) fitted to a stereomicroscope, supplied with image analysis software (ImagePro® Plus 4.5; Media Cybernetics Inc., Silver Spring, MD). Statistical analysis was conducted using IBM Statistics version 22 (SPSS, Armonk, NY, USA), by applying *t*-test to compare species samples for each variable (*P* < 0.05) and principal components analysis PCA (using varimax rotation) on the shell measurement dataset including all variables (data ln-transformed). Shell characteristics measured on 34  *L. neotropica* and 16  *L. viator* specimens were: length (SL); maximum width (SW); aperture length (AL); aperture width (AW); last spire length (LSL); spiral angle (SSA); SL/SW ratio; SL/AL ratio; and SL/LSL ratio.

Moreover, the soft parts of the snails were dissected under a stereomicroscope and the anatomy of the reproductive system analysed, to assess the morphology and measurements of the penial structures.

### Environment and climate analyses

Field studies to look for the presence of lymnaeid snails were undertaken in freshwater bodies in close proximity to the dwellings, schools and villages where infected human subjects were detected. The analysis of aspects of the ecology of the lymnaeids, such as the characteristics of their natural habitat and environment and the local climatic factors become crucial to understand the human endemic areas in question, regarding the transmission pattern [2, 26], the epidemiological scenario [2, 27] and the best strategies for diagnosis and surveys [11].

The yearly variation of the climatic factors was obtained by interpolation furnished by the closest ten surrounding meteorological stations (four and six for the transmission foci of localities A and B, respectively) from a series database of 50 years (1950–2000). The data from the agroclimatic database of the FAO Agromet Group (FAOCLIM) were used. The interpolation method applied to assess the climatic characteristics of the two aforementioned local areas in question is by nearest neighbour by using the New LocClim 1.10 software from FAO with a temporal resolution of days. This software allows for altitude and horizontal corrections. Climatic factors analysed included the maximum, mean and minimum temperatures, precipitation, potential evapotranspiration (PET), humid period (vegetation growing season), moist period (vegetation growing season), and dry period. With regard to the vegetation period, the growing season is the period during a year when precipitation exceeds half the potential evapotranspiration and is defined by Prec/PET > 0.5 [81], although for these two foci both PET > 0.5 and PET > 0.45 were analysed due to their extreme arid conditions.

The most widely applied climatic classifications were used and different appropriate climatic indices were obtained. The Koeppen climate classification is based on the concept that native vegetation is the best expression of climate [64]. According to the Koeppen classification, climate zone boundaries are selected with vegetation distribution in mind. It combines average annual and monthly temperatures and precipitation, and the seasonality of precipitation [64]. The Budyko climatic classification system is a method of categorizing climates based on the ratio of energy received to energy required to evaporate local precipitation [64]. The principles of Budyko’s classification take into account radiation characteristics, which are closely and directly correlated with the temperature in the warm season, and circulation factors, which indirectly correlated with quantitative characteristics of precipitation and moistening regime [64].

Among the variety of existing indices to quantify aridity/moisture and continentality, the aridity index of De Martonne and the continentality index of Gorczynski were obtained. The aridity index of De Martonne provides a simple way to express the ratio of precipitation to evaporation [65]. Since evaporation is rarely observed, it is a common tradition to approximate it. De Martonne’s index is defined as the ratio of the summed annual precipitation in mm and the annual mean temperature in °C +10. The continentality index of Gorczynski is a way to estimate the influence of the ocean on the local climate [65]. Gorczynski’s index depends linearly on the annual temperature amplitude (difference of monthly mean temperature of warmest and coldest month) [65].

## Results

### Molecular characterisation of lymnaeids

ITS-2, ITS-1, 16S and *cox*1 sequences reported in this study are available in the databases under the accession numbers listed in Table 1. The results revealed two lymnaeid species: *L. neotropica* and *L. viator*. Out of the 50 specimens analysed, 34 (68.0 %) were molecularly identified as *L. neotropica* and 16 (32.0 %) as *L. viator*. The latter species was detected sharing the same ecotope with *L. neotropica* in the Locality A whereas only *L. neotropica* was found in Locality B.

**Table 1:** Nuclear rDNA and mtDNA haplotype code identification for *Lymnaea neotropica and L. viator* from Argentina

Lymnaeid species	Population	rDNA ITS-2		rDNA ITS-1		mtDNA 16S		mtDNA *cox*1		Combined H nomenclature
	Locality^a^	H^b^	GenBank	H	GenBank	H^c^	GenBank	H^c^	GenBank	
			acc. no.		acc. no.		acc. no.		acc. no.	
*L. neotropica*	Locality A	1	AM412225	B^d^	KT215347	16S-A	KT226115	e^d^	KT215350	L.neo-1B,16SA,*cox*1e
*L. neotropica*	Locality B	1	AM412225	B^d^	KT215347	16S-A	KT226115	e^d^	KT215350	L.neo-1B,16SA,*cox*1e
*L. viator*	Locality A	3^d^	KT215348	C^d^	KT215349	16S-B*	KT215352	d^d^	KT215351	L.via-3C,16SB,*cox*1d

^a^Locality A: village Taton, Department of Tinogasta, province Catamarca; Locality B: village Ipizca, Department of Ancasti, province Catamarca

^b^H, haplotype

^c^Only preliminary haplotypes due to incomplete gene sequence

^d^New haplotypes for the corresponding lymnaeid species

### *Lymnaea neotropica*

#### Nuclear markers

##### rDNA ITS-2

All specimens from both localities had identical sequences (417 bp long; GC content 56.83 %). No nucleotide difference was found when comparing with the ITS-2 of the *L. neotropica* haplotype L.neo-H1 (GenBank AM412225) from the type-locality in Peru. The haplotype L.neo-H2 from Venezuela (GenBank JF514089) differed from the Argentinian sequence in the lack of one microsatellite repeat (AT) at positions 402–403 and in one mutation at position 350 of the sequence alignment of both ITS-2 haplotypes. Other Argentinian isolates of *L. neotropica* available in the GenBank database (KJ425597, 415 bp; KJ425598, 415 bp; and KJ425596, 414 bp) showed only some differences (deletions) at the 3′ end of the ITS-2, although these sequences are incomplete.

##### rDNA ITS-1

A new haplotype for *L. neotropica* was detected in the populations from localities A and B. All specimens presented the same ITS-1 of 535 bp (GC content 56.45 %). This new haplotype is here annotated as L.neo-HB. Differences with haplotype L.neo-HA from the type-locality are two insertions in the “poli-A” region at the 3′ end (positions 512 to 529), a region of 16 or 18 consecutive “A” in L.neo-HA and L.neo-HB, respectively. BLASTN results showed similarity of 98 % with other *L. neotropica* sequences obtained in Argentina available in the GenBank database (KJ425594, 528 bp; KJ425590, 528 bp; KJ425591, 527 bp; KJ425595, 525 bp; and KJ425593, 523 bp), and curiously, with one of *L. viator* (JF960165, 533 bp). Differences (indels) were again found at the 3′ end of the ITS-1 spacer of these incomplete sequences.

#### Mitochondrial markers

##### mtDNA 16S

**Fig. 2:**
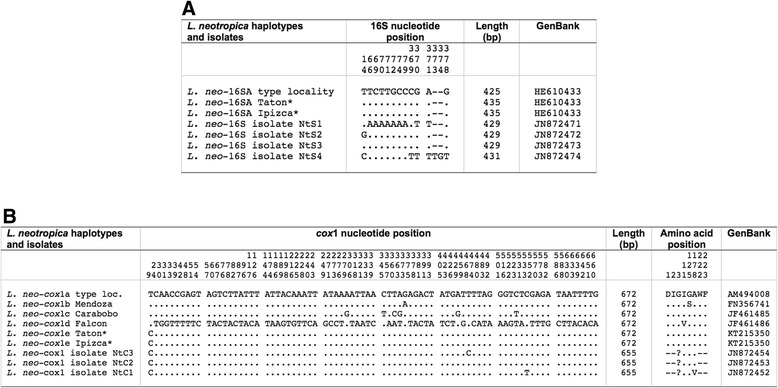
Nucleotide differences found in mtDNA sequence of *Lymnaea neotropica* populations studied and other haplotypes and isolates of the same species. **a** 16S. **b** *cox*1. Position = numbers (to be read in vertical) refer to variable positions obtained in the alignment made with MEGA 6.0.6; **.** = identical; − = indel; ? = undetermined amino acid; * = present paper. Haplotype codes only provisional due to incomplete sequences of the gene

Only one haplotype was detected. This partial sequence was 435 bp long, with a biased AT content of 69.65 %, and proved to be identical to L.neo-16SA from the type-locality also reported in the human fascioliasis endemic area of Cajamarca in Peru (GenBank HE610433). In a 439 bp long alignment of this haplotype (L.neo-16SA) with other *L. neotropica* isolates (sequences 429–431 bp long) available in GenBank, 12 variable positions were detected, most being generated by the isolate NtS1 from Argentina (GenBank JN872471), whose mutations (most of them always “A”) should be revised (Fig. 2a).

##### mtDNA *cox*1

 All specimens from localities A and B showed identical *cox*1 sequences of 672 bp (AT content 69.79 %). This sequence was compared with the four *cox*1 haplotypes described for *L. neotropica* so far and proved to be different in only one mutation at position 9 of the alignment with the previously described haplotype L.neo-*cox*1a from the type-locality (GenBank AM494008). Similarly, one mutation at position 375 was the only difference with haplotype L.neo-*cox*1b, previously reported in Mendoza, Argentina. Nucleotide and amino acid differences with all *L. neotropica* haplotypes (672 bp long) and other isolates (655 bp long) showing highly similar sequences according to BLASTN results, are detailed in Fig. 2b.

### *Lymnaea viator*

#### Nuclear markers

##### rDNA ITS-2

**Fig. 3:**
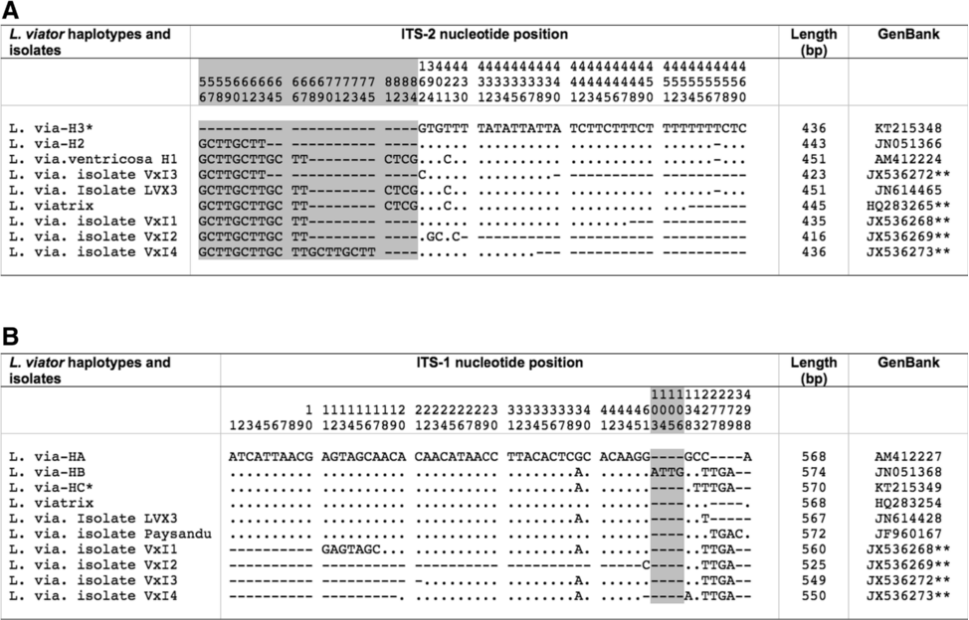
Polymorphic sites found in rDNA sequence of *Lymnaea viator* populations studied and other haplotypes and isolates of the same species. **a** ITS-2. **b** ITS-1. Position = numbers (to be read in vertical) refer to variable positions obtained in the alignment made with MEGA 6.0.6; **.**= identical; - = deletions (in microsatellite area); * = present paper; ** = incomplete sequences at the 3′ or 5′ end. Shaded area corresponds to microsatellite positions

All specimens had identical ITS-2 sequences (436 bp long; GC content 53.67 %). When compared with the available ITS-2 haplotypes of *L. viator*, the most similar to this Argentinian haplotype was L.via-H2 from Chile (GenBank JN051366), being only shorter in length due to 8 polymorphic sites corresponding to 8 indels related to a different number of repeats in the microsatellites GCTT and GCTC. These microsatellites were (GCTT)_2_, (GCTC)_1_ in Locality A, Argentina and (GCTT)_4_ and (GCTC)_1_, respectively, in Casa Blanca, Chile. The code H3 has been ascribed to this new haplotype (Table 1). Other differences with the haplotype L.via-H1 and other *L. viator* isolates showing incomplete 3′ end ITS-2 sequences are listed in Fig. 3a.

##### rDNA ITS-1

All individuals had identical ITS-1 sequences (570 bp; GC content 54.03 %). This sequence was similar to the haplotype L.via-HB from Chile (GenBank JN051368), differing in only five polymorphic sites corresponding to one mutation and one tetranucleotide repeat (ATTG). The code HC was ascribed to this new haplotype. Detailed differences with *L. viator* haplotypes HA from Argentina and HB from Chile, as well as with other *L. viator* isolates showing incomplete 5′ end ITS-1 sequences are listed in Fig. 3b.

#### Mitochondrial markers

##### mtDNA 16S

**Fig. 4:**
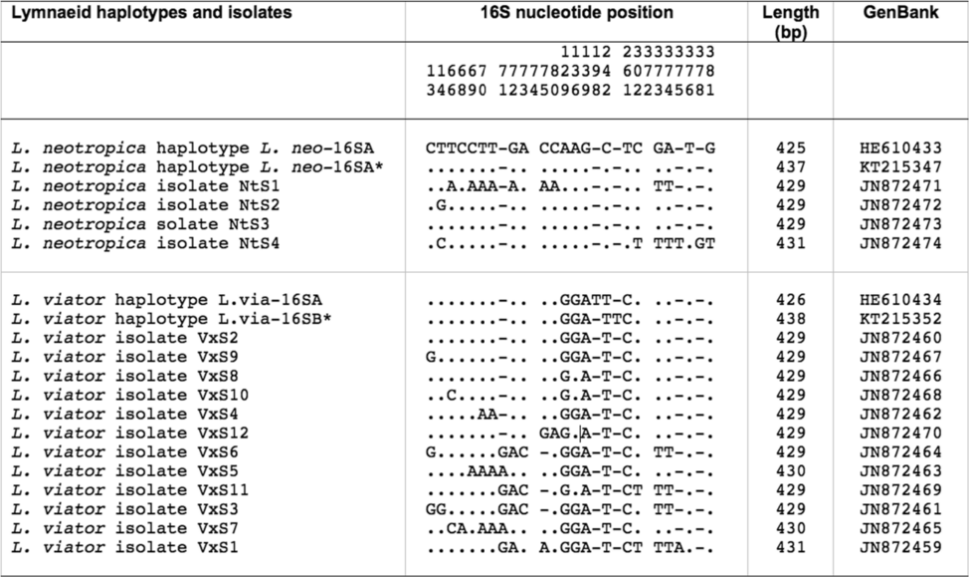
Nucleotide differences found in the mitochondrial rDNA 16S of *Lymnaea neotropica* and *L. viator* studied and other 16S haplotypes and isolates of the same species. Position = numbers (to be read in vertical) refer to variable positions obtained in the alignment made with MEGA 6.0.6; **.** = identical; − = indel; * = present paper. Haplotype codes only provisional due to incomplete sequences of the gene

A new haplotype has been detected in Locality A, for which the new code L.via-16SB is assigned. This partial sequence was 438 bp long (AT content 69.41 %). This haplotype is similar to one found in the type-locality, L.via-16SA (GenBank HE610434), which differs in only two insertion/deletions (indels). Worth noting are the very few differences detected in this fragment between specimens of *L. neotropica* and *L. viator* studied in Argentina (only five variable positions: four mutations and one indel). Therefore, a comparative sequence analysis was made including *L. neotropica* and *L. viator* haplotypes and other 16S haplotypes and isolates of the same species available in the GenBank database showing highly similar sequences according to BLASTN (Fig. 4).

##### mtDNA cox1

**Fig. 5:**
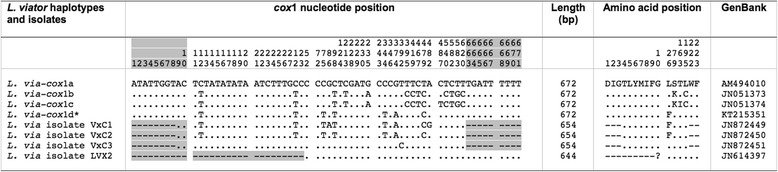
Nucleotide and amino acid differences found in the mtDNA *cox*1 of *Lymnaea viator* studied and other *cox*1 haplotypes and isolates of the same species. Position = numbers (to be read in vertical) refer to variable positions obtained in the alignment made with MEGA 6.0.6;** .** = identical; ? = undetermined amino acid; − = not sequenced; * = present paper. Shaded area corresponds to shorter fragments. Haplotype codes only provisional due to incomplete sequences of the gene

Only one nucleotide sequence was found, identical in all specimens (672 bp; AT content 69.94 %). This sequence proved to be new and the new haplotype code L.via-*cox*1d is here ascribed to it. When compared with previously described haplotypes of *L. viator cox*1a, *cox*1b, and *cox*1c, nucleotide differences ranged between 6 and 11. The haplotype L.via*-cox*1a from Rio Negro, Argentina (GenBank AM494010) appears to be the most similar. BLASTN results provided 100 % similarity only with a shorter *cox*1 fragment of *L. viatrix* (isolate VxC2; 654 bp) from Mendoza, Argentina (GenBank JN872450). Nucleotide and amino acid differences with *L. viator* haplotypes and other isolates are detailed in Fig. 5.

### Comparative sequence analysis of *L. neotropica vs L. viator*

There were 60 variable positions (29 mutations and 31 indels) in a 464 bp long ITS-2 alignment of both species and the respective haplotypes and isolates. Additionally, this marker provides microsatellites for population characterisation (Fig. 3a). For ITS-1, there were 119 variable positions (93 mutations and 26 indels) in a 580 bp long alignment. Total character differences between species were clear, and consistent, showing a range of 57 to 59 (average 57.90), with a p-distance of 0.1163 on average (or a total of 80 parsimony informative positions in their respective alignment) for species distinction. A pairwise distance matrix between ITS-2 and ITS-1 sequences of haplotypes and isolates of *L. neotropica* and *L. viator* is included in Tables 2 and 3.

**Table 2:** Pairwise distances (total character differences) between ITS-2 sequences of haplotypes and isolates of *Lymnaea neotropica* and *L. viator*

Haplotype codes and isolates	GenBank acc. no.	1	2	3	4	5	6	7	8	9	10	11	12	13	14
1. *L. neotropica* haplotype G.neo-H1	AM412225														
2. *L. neotropica* isolate Zavalla	KJ425597	0													
3. *L. neotropica* isolate Caseros	KJ425598	0	0												
4. *L. neotropica* isolate Constitucion	KJ425596	0	0	0											
5. *L. neotropica* haplotype G.neo-H2	JF514089	4	4	4	4										
6. *L. viator* haplotype L.via-H2	JN051366	13	13	13	13	16									
7. *L. viator* isolate VxI1	JX536268	13	13	13	13	16	0								
8. *L. viator* isolate VxI3	JX536272	14	14	14	14	17	1	1							
9. *L. viator* isolate LVX3	JN614465	14	14	14	14	17	1	1	2						
10. *L. viator *	HQ283265	14	14	14	14	17	1	1	2	0					
11. *L. viator* haplotype L.via-H1	AM412224	14	14	14	14	17	1	1	2	0	0				
12. *L. viator* isolate VxI2	JX536269	14	14	14	14	17	3	3	4	4	4	4			
13. *L. viator* isolate VxI4	JX536273	13	13	13	13	16	0	0	1	1	1	1	3		
14. *L. viator* haplotype L.via H3	KT215348	13	13	13	13	16	0	0	1	1	1	1	3	0	

Distances calculated in MEGA 6.0.6 using total number of differences, including transitions (ts) + transversions (tv); gaps treated as complete deletion. Haplotype codes are listed in Table 1

**Table 3:** Pairwise distances (total character differences) between ITS-1 sequences of haplotypes and isolates of *Lymnaea neotropica* and *L. viator*

Haplotype codes and isolates	GenBank acc. no.	1	2	3	4	5	6	7	8	9	10	11	12	13	14	15	16	17	18
1. *L. neotropica* isolate Zavalla	KJ425594																		
2. *L. neotropica* isolate General Lopez	KJ425590	0																	
3. *L. neotropica* L.neo-HA	AM412228	0	0																
4. *L. neotropica* isolate Caseros	KJ425591	0	0	0															
5. *L. viator* isolate San Pedro1	J5F960165	0	0	0	0														
6. *L. neotropica* isolate Caseros	KJ425595	0	0	0	0	0													
7. *L. neotropica* isolate Constitucion	KJ425593	0	0	0	0	0	0												
8. *L. neotropica* L.neo-HB	KT215347	0	0	0	0	0	0	0											
9. *L. viator ventricosa* L.via-HA	AM412227	57	57	57	57	57	57	57	57										
10. *L. viator*	HQ283254	57	57	57	57	57	57	57	57	0									
11. *L. viator* isolate LVX3	JN614428	58	58	58	58	58	58	58	58	1	1								
12. *L. viator* L.via-HB	JN051368	58	58	58	58	58	58	58	58	1	1	0							
13. *L. viator* isolate VxI1	JX536268	58	58	58	58	58	58	58	58	1	1	0	0						
14. *L. viator* isolate Paysandu	JF960167	57	57	57	57	57	57	57	57	0	0	1	1	1					
15. *L. viator* isolate VxI3	JX536272	58	58	58	58	58	58	58	58	1	1	0	0	0	1				
16. *L. viator* isolate VxI4	JX536273	59	59	59	59	59	59	59	59	2	2	1	1	1	2	1			
17. *L. viator* isolate VxI2	JX536269	58	58	58	58	58	58	58	58	1	1	0	0	0	1	0	1		
18. *L. viator* L.via-HC	KT215349	59	59	59	59	59	59	59	59	2	2	1	1	1	2	1	2	1	

Distances calculated in MEGA 6.0.6 using total number of differences, including transitions (ts) + transversions (tv); gaps treated as complete deletion. Haplotype codes correspondences are listed in Table 1

Regarding mtDNA, *cox*1 was excellent for species and populations distinction when used as nucleotide sequence, but not when translated to protein due to amino acid sequence identity of some isolates of *L. neotropica* and *L. viator*. Contrarily to the other three markers used, 16S is the only one that may create confusion if used alone for molecular differentiation of the two species. Causes for this conclusion are: the short length of the fragment used and the very low number of variable positions obtained (25, of which 19 mutations and 6 indels, in a 442 bp long alignment, 5.65 %) which do not generate a consistent separation between both species and haplotypes (Fig. 4). Total character differences were used to construct a pairwise distance matrix for 16S and *cox*1 sequences between haplotypes and isolates of *L. neotropica* and *L. viator* (Tables 4 and 5).

**Table 4:** Pairwise distances (total character differences) between 16S sequences of haplotypes and isolates of *Lymnaea neotropica* and *L. viator*

Haplotype codes and isolates	GenBank acc. no.	1	2	3	4	5	6	7	8	9	10	11	12	13	14	15	16	17	18	19	20
1. *L. neotropica* L.neo-16SA	HE610433																				
2. *L. neotropica* L.neo-16SA	KT226115	0																			
3. *L. neotropica* isolate NtS1	JN872471	8	8																		
4. *L. neotropica* isolate NtS2	JN872472	1	1	9																	
5. *L. neotropica* isolate NtS3	JN872473	0	0	8	1																
6. *L. neotropica* isolate NtS4	JN872474	5	5	9	5	5															
7. *L. viator* L.via-16SA	HE610434	5	5	13	6	5	10														
8. *L. viator* L.via-16SB	KT215352	5	5	13	6	5	10	0													
9. *L. viator* isolate VxS2	JN872460	5	5	13	6	5	10	0	0												
10. *L. viator* isolate VxS9	JN872467	6	6	14	7	6	11	1	1	1											
11. *L. viator* isolate VxS8	JN872466	4	4	12	5	4	9	1	1	1	2										
12. *L. viator* isolate VxS10	JN872468	5	5	12	6	5	10	2	2	2	3	1									
13. *L. viator* isolate VxS4	JN872462	7	7	11	8	7	12	2	2	2	3	3	4								
14. *L. viator* isolate VxS12	JN872470	5	5	11	6	5	10	2	2	2	3	1	2	4							
15. *L. viator* isolate VxS6	JN872464	10	10	12	11	10	11	5	5	5	4	6	7	7	7						
16. *L. viator* isolate VxS5	JN872463	8	8	10	9	8	13	3	3	3	4	4	5	1	5	8					
17. *L. viator* isolate VxS11	JN872469	9	9	11	10	9	8	6	6	6	7	5	6	8	6	3	9				
18. *L. viator* isolate VxS3	JN872461	11	11	13	10	11	11	6	6	6	5	7	8	8	8	1	9	4			
19. *L. viator* isolate VxS7	JN872465	9	9	12	10	9	14	4	4	4	5	5	4	2	6	9	3	10	10		
20. *L. viator* isolate VxS1	JN872459	9	9	11	10	9	8	4	4	4	5	5	6	6	6	3	7	2	4	8	

Distances calculated in MEGA 6.0.6 using total number of differences, including transitions (ts) + transversions (tv); gaps treated as complete deletion. Haplotype codes are listed in Table 1

**Table 5:** Pairwise distances (total character differences) between *cox*1 sequences of haplotypes and isolates of *Lymnaea neotropica* and *L. viator*

Haplotype codes and isolates	GenBank acc. no.	1	2	3	4	5	6	7	8	9	10	11	12	13	14	15	16
1. *L. neotropica* L.neo-*cox*1a	AM494008																
2. *L. neotropica* L.neo-*cox*1b	FN356741	1															
3. *L. neotropica* L.neo-*cox*1c	JF461485	6	7														
4. *L. neotropica* L.neo-*cox*1d	JF461486	70	71	72													
5. *L. neotropica* L.neo-*cox*1e	KT215350	0	1	6	70												
6. *L. neotropica* isolate NtC1	JN872452	1	2	7	71	1											
7. *L. neotropica* isolate NtC2	JN872453	0	1	6	70	0	1										
8. *L. neotropica* isolate NtC3	JN872454	1	2	7	71	1	2	1									
9. *L. viator ventr.* L.via-*cox*1a	AM494010	28	29	33	66	28	29	28	27								
10. *L. viator* L.via-*cox*1b	JN051373	33	34	38	70	33	34	33	34	11							
11. *L. viator* L.via-*cox*1c	JN051374	34	35	39	71	34	35	34	35	12	1						
12. *L. viator* L.via-*cox*1d	KT215351	29	30	32	65	29	30	29	28	5	12	13					
13. *L. viator* isolate VxC1	JN872449	29	30	32	65	29	30	29	28	7	14	15	2				
14. *L. viator* isolate VxC2	JN872450	29	30	32	65	29	30	29	28	5	12	13	0	2			
15. *L. viator* isolate VxC3	JN872451	29	30	34	66	29	30	29	28	1	12	13	6	8	6		
16. *L. viator* isolate LVX2	JN614397	28	29	33	66	28	29	28	27	0	11	12	5	7	5	1	

Distances calculated in MEGA 6.0.6 using total number of differences, including transitions (ts) + transversions (tv); gaps treated as complete deletion. Haplotype codes are listed in Table 1

Useful information provided by the four molecular markers applied for the distinction between *L. neotropica* and *L. viator* is detailed in Table 6. For the differentiation between these two species, ITS-1 is the marker with a higher resolution and 16S as the worst. For intraspecific analyses, *cox*1 appears to be the best marker and ITS-1 the worst. The low resolution of the 16S gene fragment for species distinction is worth mentioning. For instance, 16S distance estimation using as substitution model total nucleotide differences (ts + tv) between *L. neotropica* and *L. viator* ranged between 4 and 14 *vs* 0–9 and 0–10 within *L. neotropica* and *L. viator* sequences, respectively (Table 6).

**Table 6:** Average intra- and interspecific genetic distances (nt) for *Lymnaea neotropica* and *L. viator*

Groups analysed	ITS-2	ITS-1	16S	*cox*1
*L. neotropica vs L. viator*	15.15 (0.0354)	57.90 (0.1163)	8.56 (0.0202)	35.20 (0.0554)
*L. neotropica*	1.60 (0.0040)	0 (0)	4.33 (0.0102)	19.61 (0.031)
*L. viator*	1.39 (0.0035)	0.87 (0.0017)	4.31 (0.0102)	7.53 (0.012)

The number of base differences per site from averaging over all sequence pairs between and within groups are shown. Distances calculated using as substitution model total nucleotide differences (ts + tv) and p-distance (in parentheses) methods. Fewer than 5 % alignment gaps, missing data and ambiguous bases were allowed at any position (all positions with less than 95 % site coverage were eliminated)

### Phylogenetic results

ML, NJ and Bayesian phylogenetic methods yielded similar tree topologies for the combined dataset used (ITS-1 + ITS-2; 1417 bp).

**Fig. 6:**
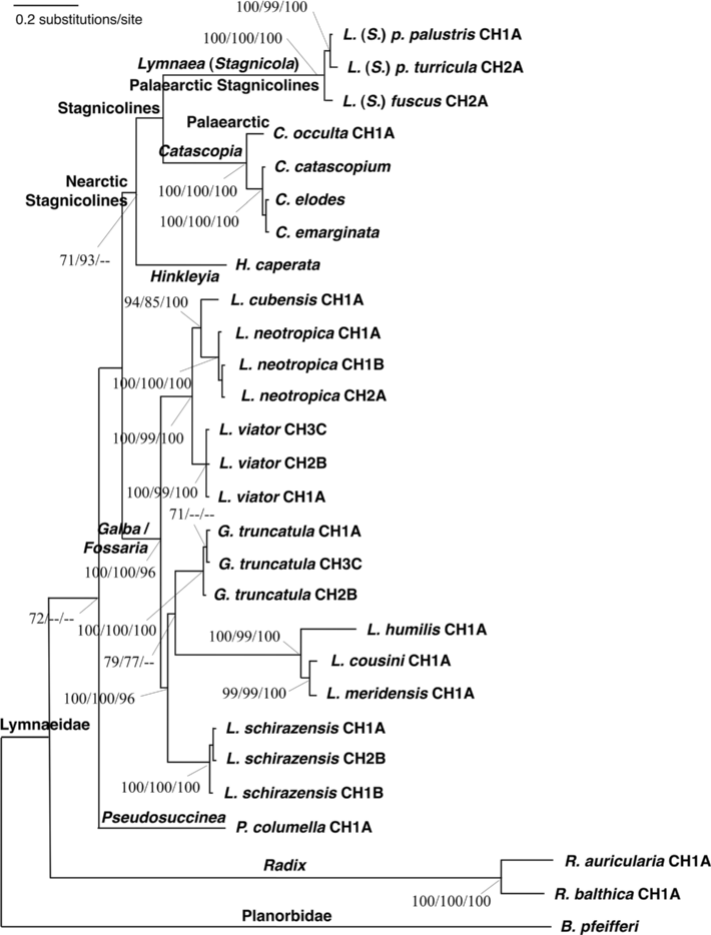
Phylogenetic tree (Log-Likelihood = −11,597.42) of lymnaeid species studied based on maximum-likelihood (ML) estimates (Ts/Tv = 1.1445; Gamma = 0.6637). Scale-bar indicates the number of substitutions per nucleotide position. Node support values with 1,000 bootstrap replicates based on a/b/c: a: bootstrap with Neighbour-Joining search (only values > 70 shown); b: bootstrap with “Fast” stepwise-addition search (only values > 70 shown); c: Bayesian posterior probability (BPP) (only values > 95 shown)

The ML tree obtained (−Ln = 11,597.42) (Fig. 6) including the new haplotypes, together with published GenBank sequences of related species, showed that *L. neotropica* and *L. viator*, together with *Lymnaea cubensis*, had strong support as sister species and constituted a monophyletic clade inside the *Galba/Fossaria* group. Other members of this group, i.e. *G. truncatula*, *L. humilis*, *L. cousini*, *L. meridensis* and *L. schirazensis* clustered together in another monophyletic clade. *Pseudosuccinea columella* appeared basal to the two large groupings, namely stagnicolines (including both Palaearctic and Nearctic species) and the *Galba/Fossaria* clade which comprised the main host species of *F. hepatica. Radix* spp. clustered independently in a branch basal to all lymnaeids in this analysis.

### Morphometric study of the lymnaeid species

The coexistence of *L. neotropica* and *L. viator* in Locality A furnished the possiblity for a comparative shell morphology study to assess whether these species may be morphometrically distinguished within the same endemic area.

**Fig. 7:**
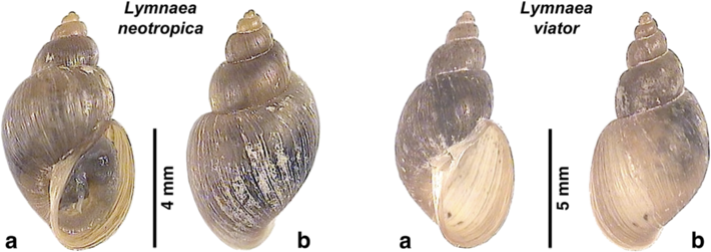
Shell of *Lymnaea neotropica* and *L. viator* from Locality A (Taton-Rio Grande). **a** Ventral views. **b** Dorsal views

The shell of both species is brownish to light brown, thin-walled, elongated conical, usually with four regular convex whorls (up to 5.5 whorls in longer specimens). The spire is more or less pointed or short with a rather obtuse apex. Growth lines and umbilicus are slightly pronounced. The spiral sculpture is very faint and not easily seen. The aperture is oval and almost round, with a thin peristome. The shape of the shell of *L. neotropica* and *L. viator* (Fig. 7) is very similar, both with a similar whorl number, although the specimens of *L. viator* may sometimes appear somewhat more slender (compare in Fig. 7).

**Fig. 8:**
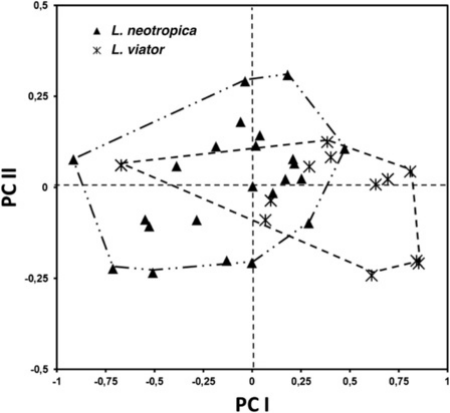
Principal components analysis (PCA) plot based on shell measurement datasets of *Lymnaea neotropica* and *L. viator* (variables ln-transformed). The samples are projected onto the first (PCI) and second (PCII) principal components explaining 65 and 18 % of the variation, respectively

The comparative data for shell measurements and shell ratios (Additional file 1: Table S1) show that the shell length/shell maximum width ratio overlaps in the two species and hence the variability of this parameter cannot be used for their differentiation. A similar problem appears evident in relation to all other shell parameters, except for the shell length which not only reaches an apparently higher length maximum (10.85 *vs* 9.18 mm) but also a greater shell length mean (9.21 *vs* 7.49 mm) in *L. viator*. The results of the statistical comparisons by means of *t*-test are included in Additional file 1: Table S1. Only the shell spiral angle and the three shell ratios furnished data exhibiting statistical significant differences between the two species. The principal components analysis furnished a graph in which data of the two species overlap (Fig. 8).

At anatomical level, only the preputium/penis-sheath length ratio allowed for a species differentiation. This ratio proved to be of 1.14 in *L. neotropica* and 2.78 in *L. viator*.

### Climatic characteristics and snail habitats

**Fig. 9:**
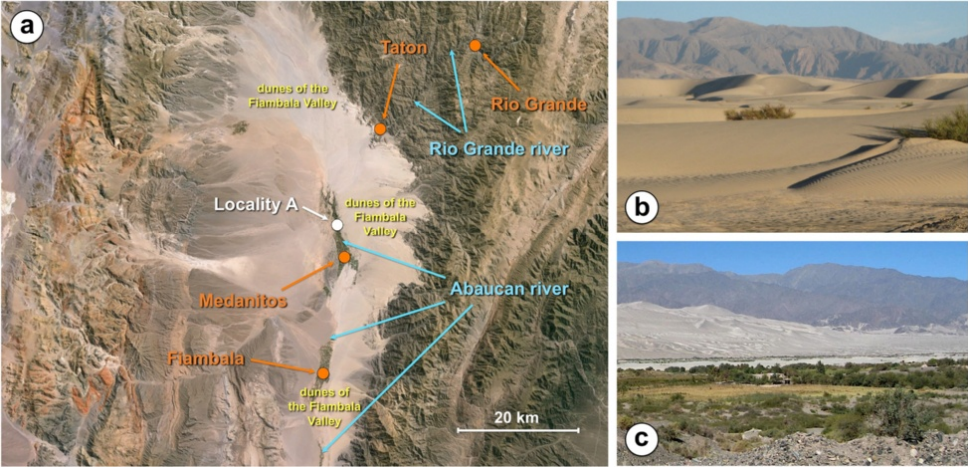
Transmission focus of Locality A. **a** Geographical image showing Locality A where lymnaeid snails were collected, on the way from the small village of Medanitos to the small villages of Taton and Rio Grande (image from Google Earth taken at 90 km altitude; image Landsat, US Department of State Geographer, 2016); note desert surroundings of sand dunes of the Fiambala Valley and high mountain chains at the east and west of the desert. **b** Aspect of sand dunes surrounding Locality A. **c**: Overview of Locality A with sand dunes and eastern mountain chain in the background

The results of the climatic analyses of the localities A and B, according to the different climate classification systems and the different climatic indices used, are listed in Table 7. The results of the analyses by the climate classifications of Koeppen and Budyko, as well as by the aridity index of De Martonne, allow to highlight the extreme desertic-arid environmental characteristics surrounding Locality A (Fig. 9) and the slightly less extreme conditions of semiaridity-aridity of those surrounding Locality B. The influence of the ocean on the local climate estimated by the continentality index of Gorczynski indicates small differences between the two localities situated at different altitudes and west and east of the same mountain chain.

**Table 7:** Climatic characteristics of the Locality A (Taton) and Locality B (Ipizca) according to the different climate classification systems and the different climatic indices used

Transmission foci	Locality A (Taton)	Locality B (Ipizca)
Coordinates	27°29′52.62″S, 67°35′ 53.51″W	28°49′08.83″S, 65°32′20.00″W
Altitude (m)	1,630	944
Koeppen class	BWk	BSh
	B = Arid Climate	B = Arid Climate
	D = Desert	S = Steppe
	k = Cold	h = Hot
Budyko climate	Desert	Semiarid
Radiation index of dryness	9.236	3.009
Budyko evaporation (mm/year)	151	429
Budyko runoff (mm/year)	0	19
Budyko evaporation (%)	100	95.8
Budyko runoff (%)	0	4.2
Aridity	Arid	Semiarid
Aridity index	0.09	0.25
Moisture index (%)	-91	-75
De Martonne index	6	15
Precipitation deficit (mm/year)	1,547	1,333
Gorczynski continentality index	38.5	34.6

**Fig. 10:**
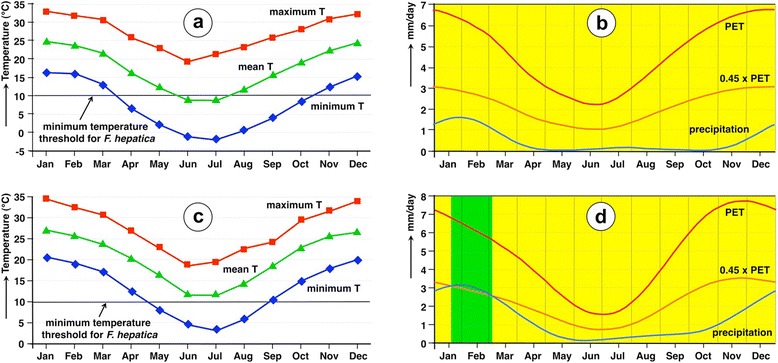
Yearly variation of monthly values of climatic factors during the period 1950–2000. **a**, **b** Locality A (Taton-Rio Grande). **c**, **d** Locality B (Ipizca). Left column graphs (**a**, **c**) correspond to yearly variation of temperatures (data in °C): red squares and line = mean maximum temperature (MMT); green triangles and line = mean environmental temperature (MET); blue diamonds and line = mean minimum temperature (MmT). Right column graphs (**b**, **d**) correspond to yearly variation of precipitation and potential evapotranspiration (data in mm/day) to show differences between the rainy and dry periods: blue line = precipitation; red line = potential evapotranspiration (PET); pink line = 0.45 × PET; dark blue areas = humid period (vegetation growing season - no one appears); green areas = moist period (vegetation growing season); yellow areas = dry period

In Locality A, the maximum, mean and minimum daily temperatures show a marked monoseasonality, with a peak in January and a decrease up to the minimum values in June-July (Fig. 10a). Mean temperature values are only lower than the minimum temperature threshold of 10 °C known for *F. hepatica* during the period of June-July.

In Locality B, the maximum, mean and minimum daily temperatures show a similar seasonality, although with somewhat higher temperatures (Fig. 10c). The mean temperature values are higher than the minimum temperature threshold of 10 °C known for *F. hepatica* throughout the whole year.

The analysis of the yearly variation of precipitation and potential evapotranspiration shows the very low availability of rainfall in both localities, with a small peak in January-February (slightly higher in Locality B) and almost inexistent precipitation during the period from April-May to October-November (Fig. 10b, d). Worth emphasising is the extension of dryness throughout the year in both localities, so that only in Locality B a very short moist period appears from mid-January to beginning of March when PET > 0.45 is applied (none when using PET > 0.5) (Fig. 10d).

These results indicate that the transmission foci of localities A and B where lymnaeid snails were collected are indeed isolated environments presenting water availability depending only on the rivers flowing from the neighbouring mountain chain. These biotopes appropriate for lymnaeid snail survival are confined to lateral river side floodings and small man-made irrigation systems in both Locality A (Additional file 2: Figure S1 a-g) and Locality B (Additional file 2: Figure S1 h-j). In Locality A, lymnaeid populations may reach high densities on mud and neighbouring superficial water borders in the adequate season (Additional file 3: Figure S2).

## Discussion

### DNA characterisation of lymnaeid vectors

In *L. neotropica*, the two populations of localities A and B were characterised by having identical sequences for the four DNA markers used, including two new haplotypes for ITS-1 and *cox*1 (Table 1). The new ITS-1 (L.neo-HB) haplotype is worth nothing, given the uniformity so far detected at the level of the nuclear rDNA spacers in this species throughout. The only previously known ITS-1 haplotype (L.neo-HA) has been reported from Lima (type-locality) [19] and Cajabamba [51], both in Peru, and also in Perdriel, Departamento de Lujan de Cuyo, Mendoza, Argentina [50]. The other new haplotype in a mtDNA marker (L.neo-*cox*1e) differs from the *cox*1 haplotypes described in *L. neotropica* in other countries such as Peru, Venezuela and also Argentina. In the latter country, this new L.neo-*cox*1e haplotype only shows identity with the *cox*1 sequence of the isolate NtC2 (GenBank: JN872453) previously reported from the same lymnaeid species in Mendoza [82], although unfortunately the fragment available from that isolate is shorter (655 bp *vs* 672 bp).

The haplotypes found in the other two markers in *L. neotropica*, one in rDNA ITS-2 (L.neo-H1) and the other in mtDNA 16S (L.neo-16SA), were already reported from another area of Argentina, namely in Perdriel [50]. These two ITS-2 and 16S haplotypes were also detected in Peru, in the type-locality besides Lima [19] and also in Cajamarca [51].

In *L. viator*, four new haplotypes were found in ITS-2, ITS-1, 16S and *cox*1 in Locality A (Table 1). Detecting a new haplotype in every marker studied in a single lymnaeid population is really surprising. Previously described haplotypes and isolate sequences of the same DNA markers of *L. viator* have been cited from different localities of Chile, Argentina and Uruguay (see Figs. 3, 4 and 5).

The analysis of sequences of *L. neotropica* and *L. viator* available in the GenBank database shows that several sequences are duplicated. For instance, at ITS-2 level, two sequences of *L. viator* from Rio Negro, Argentina (HQ283265 and JN614465), are identical to the sequence of *L. viator ventricosa* H1 (AM412224) from the type-locality (Rio Negro) [19]. Similarly, another ITS-1 sequence (HQ283254) is identical to the sequence of *L. viator ventricosa* HA (AM412227) from the same type-locality (Rio Negro) [19]. Similar situations are also found in mtDNA sequences, e.g. a 16S sequence of *L. neotropica* (JN872473) which is identical to that of the haplotype *L.neo-*16SA (HE610433) from the type-locality of the species [51]. Also another *L. viator cox*1 sequence (JN614397) from Rio Negro is identical to that of *L.*via*-cox*1a (AM494010) from the same type-locality [19]. Additionally, two sequences deposited in the databases as belonging to *L. viator* ITS-1 (JF960165 and JF960166) actually belong to *L. neotropica*, although species ascription was corrected in the publication [83].

### Phylogenetic analysis

Although many attempts to divide the *Galba*/*Fossaria* group of lymnaeids into different genera have been made since long ago, proposals have never been unanimously accepted [84–89]. Moreover, molecular analyses on genetic distances by appropriately considering the different weights of the different nucleotide differences and phylogenetic assessments by different tree reconstruction methods offer different results which do not allow for a clear taxonomic arrangement of the *Galba*/*Fossaria* species so far. Such a lack of molecular systematics consistency appears using either mitochondrial DNA markers [90] or nuclear ribosomal DNA markers [20], as well as both markers together [18].

The phylogenetic tree obtained in the present study reinforces the arguments against including the American native species *L. neotropica*, *L. viator* and *L. cubensis* within the genus *Galba*, which is defined by the type-species *G. truncatula* of Euro-Asian origin. Therefore, we have kept the two lymnaeid species found in the Catamarca province within the genus *Lymnaea* (*sensu lato*) throughout.

### Morphometric characterisation of *L. neotropica* and *L. viator*

In lymnaeids, the interspecific morphological and anatomic uniformity exhibited by numerous species gives usually rise to serious difficulties in specimen identification, sometimes even impeding it [17–19]. Moreover, intraspecific variation of shell shape is particularly well marked within lymnaeids depending on environmental conditions. Such specimen identification problems are well known in species of the *Galba*/*Fossaria* group [18].

Within the *Galba*/*Fossaria* group, there are species which show high transmission capacity and others non-susceptible to *F. hepatica*, such as *L. schirazensis* [18]. Moreover, there are *Galba*/*Fossaria* species clearly related to human fascioliasis endemic areas because of their anthropophilic ecology and behaviour, whereas other species do usually inhabit biotopes frequented by livestock [38, 51]. There is therefore an applied interest in species differentiation when dealing with an endemic area and also their geographical distribution throughout a country [28].

The morphometric study performed to assess whether *L. neotropica* and *L. viator* could be morphologically distinguished in Locality A has shown an overlap in all shell features, except for the maximum length that may be greater in *L. viator* (Additional file 1: Table S1). However, no definitive conclusion on this potentially differing feature depending on growth can be reached through measurements obtained from specimens collected in nature. Unfortunately, the single absolute measurement of the shell which showed a statistical capacity for species differentiation was the shell spiral angle. Despite its usefulness for shell description, this angle is known to provide a useless marker for species differentiation because (i) there is no exact way to measure it [78] and (ii) it shows large intraspecific and even intrapopulational variability in lymnaeids [84]. The statistical analysis suggested that the three shell ratios might be helpful for species differentiation. However, regarding SL/SW, the existence of slender shells together with somewhat stouter ones was already emphasised in the type-locality of *L. viator* [91]. Concerning SL/AL and SL/LSL, ranges overlap completely and means are almost equal for both species (see Additional file 1: Table S1). Even using the combination of all shell parameters, the identification of a specimen is subject to a high risk of confusion, as shown by the results of the principal components analysis (see Fig. 8). The only species difference was the preputium/penis-sheath length ratio, close to 1 in *L. neotropica vs* close to 3 in *L. viator*. Unfortunately, obtaining this ratio needs a time-consuming anatomical dissection and malacological expertise which may not be available among public health responsibles working in the field. Moreover, a variability in this ratio of 1.1–3.9 in *L. neotropica* has been found throughout its geographical distribution [19, 82, 91, 92].

Additionally, the two species appear to coexist in the same water collections in the aforementioned locality, reiterating a similar situation of overlap of these two species in many places of the Argentinian province of Mendoza [93]. A similar phenomenon leading to confusion of the lymnaeid species due to the impossibility of distinguishing between two species and the repercussions for the disease transmission has recently been evoked in the case of *G. truncatula* and *L. schirazensis* [51].

### Climatic and environmental characterisation of the lymnaeid habitats

The extreme desertic-arid environmental characteristics surrounding Locality A and the slightly less extreme conditions of semiaridity-aridity of those surrounding Locality B, as well as the very low yearly precipitation in both localities, are surprising and very different from the typical environmental characteristics surrounding transmission foci of fascioliasis. In the two localities studied, lymnaeid snail habitats are confined to lateral river side floodings and small man-made irrigation systems.

In the aforementioned extreme situations, water availability only depends on water from the rivers and hence almost exclusively from rainfall, snow melting and deglaciation in the mountains where the rivers originate. A similar fascioliasis transmission dependence on river water coming from rainfall (monzoon) in the neighbouring mountains (Himalaya) in a markedly very large dry area (Punjab province) has recently been highlighted in Pakistan [6]. In the Argentinian areas studied here, the data indicate a concentration of all disease transmission factors in small areas where humans and animals go for water supply, vegetable cultures and livestock farming. Such a concentrated transmission focus such as in Locality A reminds the epidemiological characteristics of the transmission foci of schistosomiasis in oases of the Sahara Desert in Africa [94].

In both localities studied, a seasonal transmission may be expected depending on the timely overlap of the appropriate temperature and water availability from the rivers. Such a seasonality is known in endemic areas following the “valley pattern” of fascioliasis transmission known in other Andean countries [26, 95]. *Lymnaea neotropica* has already been linked to human fascioliasis hyperendemic areas following the “valley pattern”, as in the valley of Cajamarca, Peru [51]. *Lymnaea viator* was so far related to areas where human infection concerning a sporadic case or a few patients has been reported in Argentina [61, 96], because recent knowledge suggests that its link to human endemic areas in Chile may involve a confusion with *G. truncatula* [38].

## Conclusions

The finding of an unusual high number of DNA haplotypes in both lymnaeid vector species and the extreme climatic factors (almost total lack of rainfall and moisture) unsuitable for *F. hepatica* and freshwater lymnaeid snail development, demonstrate that the transmission foci are completely isolated. Consequently, it may be concluded that both the lymnaeid species and *F. hepatica* have reached both localities by means of livestock introduction. Moreover, DNA differences from other populations of *L. neotropica* and *L. viator* previously studied in Argentina, suggest that this introduction phenomenon was independent from the spreading movements which allowed these two lymnaeid species to expand throughout the country.

## Additional files

Additional file 1: Table S1. Lymnaeid shell measurements for natural populations of morphologically similar species, *Lymnaea viator* and *Lymnaea neotropica* from the present study and *Galba truncatula*, including *t*-test statistical comparison of variables of *L. viator* vs *L. neotropica* in Locality A. (PDF 67 kb) Additional file 2: Figure S1. Freshwater habitats of lymnaeids. **a-g** Locality A: in villages of Taton and Rio Grande. **h**-**j** Locality B: in the neighbourhood of Ipizca. **b** Note general aridity. **c** Sand dunes in the background. **b**, **d** Closeness to human dwellings. **g** Lymnaeids present on mud. **i** Small artificial dyke on the river. (PDF 448 kb) Additional file 3: Figure S2. High density of lymnaeids on mud and freshwater border in Locality A (village of Taton). (PDF 415 kb)

## Abbreviations

16S rDNA: ribosomal RNA coding large subunit gene (16S) of the mitochondrial genome
COX1: cytochrome *c* oxidase subunit 1, amino acid sequence
*cox*1: cytochrome *c* oxidase subunit 1, nucleotide sequence
FAOCLIM: agroclimatic database of the FAO Agromet Group
H: haplotype
indel: insertion/deletion
ITS-1: first internal transcribed spacer
ITS-2: second internal transcribed spacer
MET: mean environmental temperature
MMT: mean maximum temperature
MmT: mean minimum temperature
mtDNA: mitochondrial DNA
PET: potential evapotranspiration
rDNA: nuclear ribosomal DNA
ts: transition
tv: transversion
