# Supplementary material for: Human fascioliasis endemic areas in Argentina: multigene characterisation of the lymnaeid vectors and climatic-environmental assessment of the transmission pattern
Source: Parasit Vectors. 2016 May 27;9:306. doi: 10.1186/s13071-016-1589-z (PMC4882814; doi:10.1186/s13071-016-1589-z)
Supplement: Additional file 1: Table S1. — Lymnaeid shell measurements for natural populations of morphologically similar species, Lymnaea viator and Lymnaea neotropica from the present study and Galba truncatula, including t-test statistical comparison of variables of L. viator vs L. neotropica in Locality A. (PDF 67 kb) [file 13071_2016_1589_MOESM1_ESM.pdf]

Additional file 1: Supplementary Table S1 Lymnaeid shell measurements for natural populations of morphologically similar species, *Lymnaea viator* and *Lymnaea neotropica* from the present study and *Galba truncatula*, including *t*-test statistical comparison of variables of *L. viator* vs *L. neotropica* in Locality A

	Lymnaea viator		*t*-test comparison		*Lymnaea neotropica*				*Galba truncatula*			
	Locality A, Taton, Tinogasta, Catamarca, Argentina (n = 16)		*L. neotropica* vs *L. viator* in Locality A		Locality A, Taton, Tinogasta, Catamarca, Argentina (n = 34)		Rio Rimac, Peru (type locality) (type specimens)		Albufera Valencia, Spain (n = 30)		Oued Tiout, Morocco (n = 30)	
Shell characteristics	Min. and max. extreme values	Mean (± SD)	*t* value [$t_{(48)}$]	P value	Min. and max. extreme values	Mean (± SD)	Min. and max. extreme values	Mean (± SD)	Min. and max. extreme values	Mean (± SD)	Min. and max. extreme values	Mean (± SD)
Length (SL)	5.62-10.85	9.21 (± 1.52)	-3.985	0.000	5.52-9.18	7.49 (± 0.89)	5.89-8.74	7.13 (± 1.20)	6.79-9.33	7.73 (± 0.64)	6.57-8.29	7.58 (± 0.48)
Maximum width (SW)	3.11-5.79	4.88 (± 0.84)	-3.316	0.002	3.13-5.01	4.10 (± 0.47)	3.29-4.56	3.95 (± 0.54)	3.70-4.90	4.29 (± 0.29)	3.79-4.60	4.26 (± 0.22)
Aperture length (AL)	2.97-6.05	4.73 (± 0.83)	-3.935	0.000	3.14-6.64	3.85 (± 0.40)	2.90-4.53	3.75 (± 0.64)	3.30-4.07	3.58 (± 0.20)	3.29-4.29	3.86 (± 0.28)
Aperture width (AW)	1.90-3.31	2.73 (± 0.42)	-3.907	0.001	1.88-2.74	2.28 (± 0.23)	1.97-2.99	2.46 (± 0.44)	2.05-2.72	2.40 (± 0.17)	2.40-3.15	2.77 (± 0.21)
Last spire length (LSL)	4.52-8.31	6.99 (± 1.06)	-4.149	0.000	4.42-8.93	5.76 (± 0.60)	4.47-6.78	5.58 (± 0.91)	5.28-6.80	5.78 (± 0.40)	5.19-11.09	6.05 (± 1.02)
Spiral angle (SSA)	41.30°-54.91°	47.04° (± 4.35)	-0.988	0.331	33.02°-54.68°	45.09° (± 5.92)	38.64°-46.99°	42.73° (± 2.44)	33.94°-59.26°	42.73° (± 5.34)	40.91°-61.66°	50.77° ± 5.37)
SL/SW ratio	1.67-2.02	1.89 (± 0.10)	-1.917	0.065	1.69-1.98	1.83 (± 0.08)	1.69-1.92	1.80 (± 0.07)	1.55-1.96	1.80 (± 0.09)	1.64-2.02	1.78 (± 0.08)
SL/AL ratio	1.78-2.11	1.95 (± 0.10)	-0.201	0.842	1.75-2.10	1.94 (± 0.10)	1.76-2.03	1.91 (± 0.09)	1.84-2.38	2.16 (± 0.14)	1.84-2.14	1.97 (± 0.07)
SL/LSL ratio	1.24-1.37	1.31 (± 0.04)	-1.102	0.279	1.23-1.38	1.30 (± 0.04)	1.21-1.38	1.28 (± 0.05)	1.25-1.47	1.34 (± 0.05)	0.69-1.36	1.27 (± 0.11)

Range include minimum and maximum extremes, with mean (± standard deviation SD in parentheses). Measurements in mm (except spiral angle in degrees). n = number of specimens measured.
