# Supplementary material for: Human fascioliasis endemic areas in Argentina: multigene characterisation of the lymnaeid vectors and climatic-environmental assessment of the transmission pattern
Source: Parasit Vectors. 2016 May 27;9:306. doi: 10.1186/s13071-016-1589-z (PMC4882814; doi:10.1186/s13071-016-1589-z)

Additional file 2: Supplementary Fig. S1

Freshwater habitats of lymnaeids. **a-g** Locality A: in villages of Taton and Rio Grande; **h-j** Locality B: in the neighbourhood of Ipizca. **b** Note general aridity. **c** Sand dunes in the background. **b, d** Closeness to human dwellings. **g** Lymnaeids present on mud. **i** Small artificial dyke on the river.

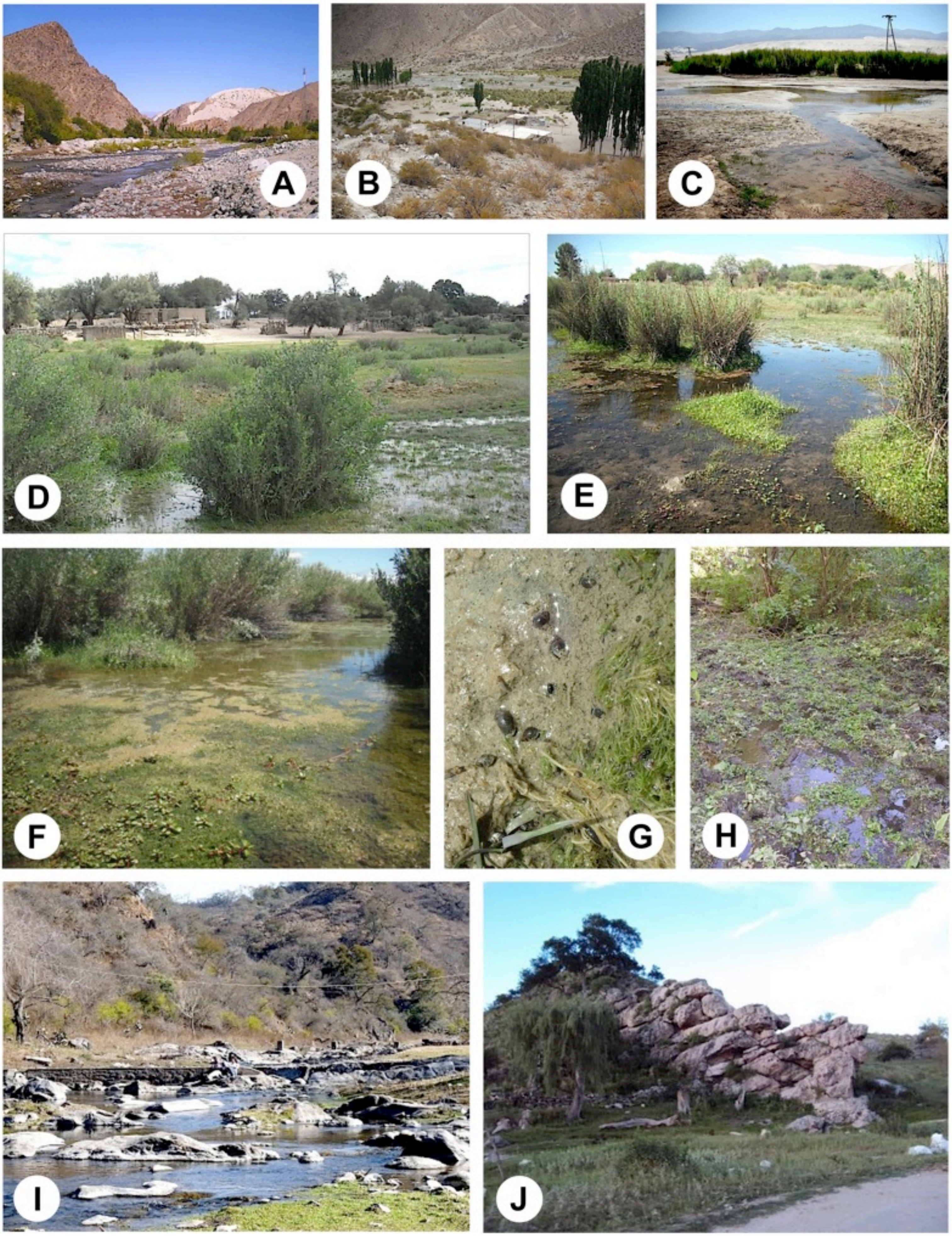

Supplement: Additional file 2: Figure S1. — Freshwater habitats of lymnaeids. a-g Locality A: in villages of Taton and Rio Grande. h-j Locality B: in the neighbourhood of Ipizca. b Note general aridity. c Sand dunes in the background. b, d Closeness to human dwellings. g Lymnaeids present on mud. i Small artificial dyke on the river. (PDF 448 kb) [file 13071_2016_1589_MOESM2_ESM.pdf]
