# Supplementary material for: Human fascioliasis endemic areas in Argentina: multigene characterisation of the lymnaeid vectors and climatic-environmental assessment of the transmission pattern
Source: Parasit Vectors. 2016 May 27;9:306. doi: 10.1186/s13071-016-1589-z (PMC4882814; doi:10.1186/s13071-016-1589-z)

Additional file 3: Supplementary Fig. S2

High density of lymnaeids on mud and freshwater border in Locality A (village of Taton).

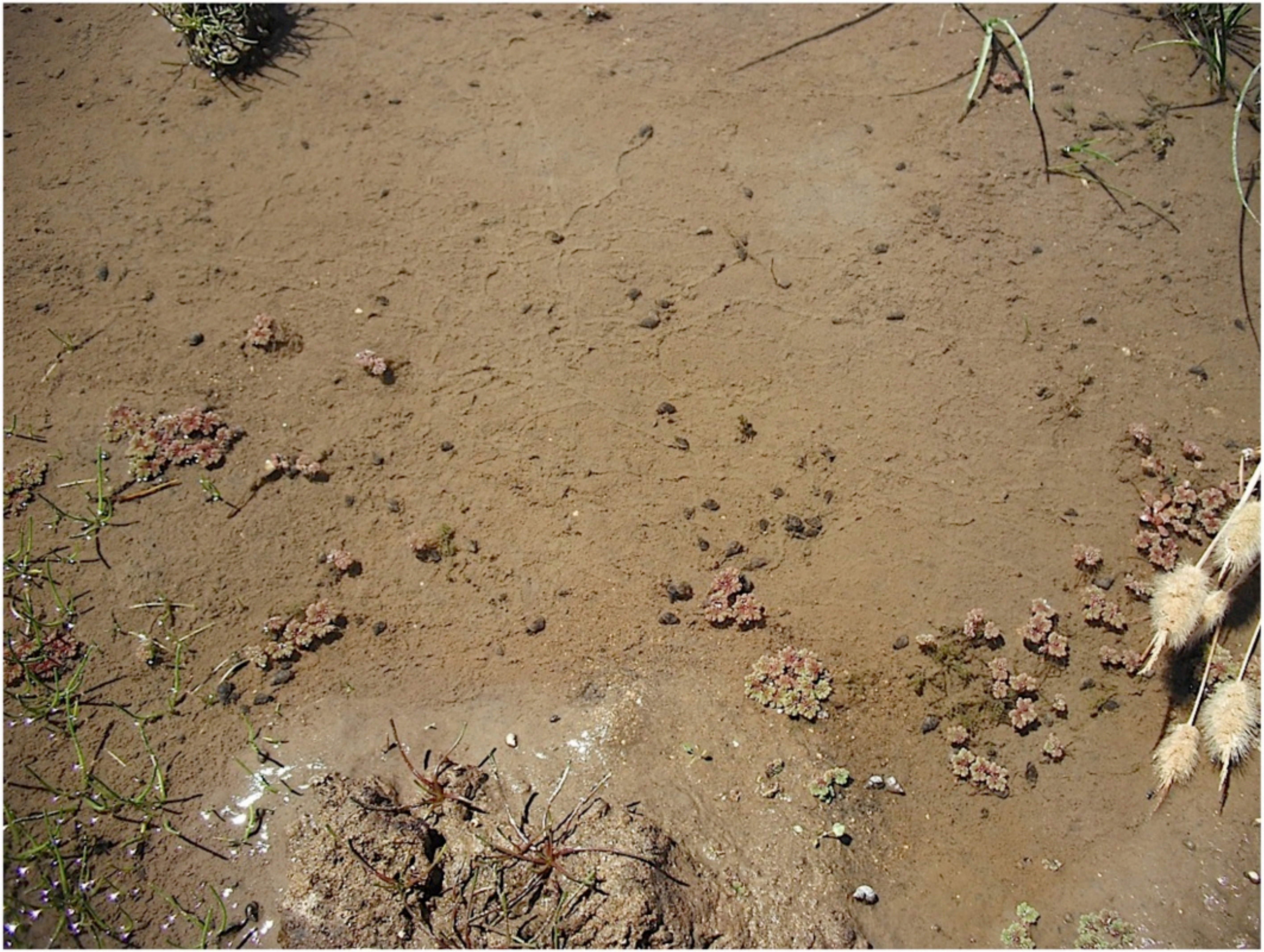

Supplement: Additional file 3: Figure S2. — High density of lymnaeids on mud and freshwater border in Locality A (village of Taton). (PDF 415 kb) [file 13071_2016_1589_MOESM3_ESM.pdf]
